# Joint associations of physical activity and screen time with overweight among japanese adults

**DOI:** 10.1186/1479-5868-8-131

**Published:** 2011-11-30

**Authors:** Yung Liao, Kazuhiro Harada, Ai Shibata, Kaori Ishii, Koichiro Oka, Yoshio Nakamura, Takemi Sugiyama, Shigeru Inoue, Teruichi Shimomitsu

**Affiliations:** 1 Graduate School of Sport Sciences, Waseda University, Nakamura Laboratory 2-579-15 Mikajima Tokorozawa, Saitama 359-1192, Japan; 2 Japan Society for the Promotion of Science, Sumitomo-Ichibancho Bldg., 6 Ichibancho, Chiyoda-ku, Tokyo 102-8471, Japan; 3 Faculty of Sport Sciences, Waseda University, 2-579-15 Mikajima Tokorozawa, Saitama 359-1192, Japan; 4 Baker IDI Heart and Diabetes Institute, Level 4, 99 Commercial Road, Melbourne VIC 3004, Australia; 5 Department of Preventive Medicine and Public Health, Tokyo Medical University, 6-1-1, Shinjuku-ku, Tokyo, 160-8402, Japan

## Abstract

**Background:**

Although both insufficient physical activity (PA) and high screen time (ST) are independent risk factors for obesity, how the combination of sufficient/insufficient PA and high/low ST could increase obesity risk among the adult population of Japan is not known. This study examined joint associations of PA and ST with overweight among Japanese adults.

**Methods:**

An Internet-based survey collected data on height, weight, self-reported time spent in PA and ST, and sociodemographic variables from 2832 adults. Respondents were categorized into sufficient PA/low ST, sufficient PA/high ST, insufficient PA/low ST, or insufficient PA/high ST categories as per public PA guidelines and the median of ST. Logistic regression analysis examined the odds ratios (OR) of being overweight (body mass index, ≥ 25 kg/m^2^) according to the categories of PA and ST.

**Results:**

In comparison with the sufficient PA/low ST category, participants in the insufficient PA/high ST category were significantly more likely overweight (OR, 1.48; 95% confidence interval [95%CI), 1.14, 1.93) after adjusting for sociodemographic variables. A significantly higher OR for overweight (including obesity) among insufficient PA/high ST category was also observed in men, but no significant association was found in women.

**Conclusions:**

Both insufficient PA and prolonged ST contribute to overweight and obesity among Japanese adults. Public health initiatives addressing obesity in Japan need to consider both promoting PA and reducing ST, especially in men.

## Background

Overweight and obesity increase the risk of developing chronic diseases including cardiovascular disease, hypertension, type 2 diabetes, and certain types of cancer [1,2]. Physically inactive lifestyles are considered to play important roles in the current obesity epidemic [3]. Research has consistently shown that physical activity (PA) is inversely associated with obesity measures [4,5]. Time spent in sitting (sedentary behavior) is also known to be associated with increased risk of obesity, independent of participation in PA [6,7].

Drawing on these research findings, an Australian study has examined the joint association of PA and sedentary behavior with obesity [8]. The study found that those who met PA guideline recommendations but reported prolonged sedentary time and those with insufficient PA and lower sedentary time had similarly higher likelihood of being overweight compared with those who conducted sufficient PA and were low in sedentary time [8]. However, the combined effect of PA and sedentary behavior on obesity is not known in other countries. Japan offers a unique research opportunity in this context. Although the prevalence of obesity is relatively low in Japan compared with Western countries, it is increasing steadily [9]. In addition, partly because of easily available media-related technologies, television/video viewing and Internet use are highly prevalent and increasing among adults [10]. Since computer and Internet use has been found associated with adult overweight and obesity [11], it is of interest to examine the health impact of screen-based sedentary behavior in the presence (and absence) of PA. This study examined the joint associations of PA and screen-based sedentary behavior with overweight among Japanese adults.

## Methods

### Participants

Data for this study came from an Internet-based cross-sectional survey in 2009. A total of 9418 adults were randomly selected from the database of a Japanese research service company (with approximately 264,000 registrants) and received invitation e-mails. Of these, 3000 individuals responded to the survey. Detailed methods and procedures have been reported elsewhere [12]. This study received prior approval from the Ethics Committee of Waseda University.

### Outcome variable

The outcome variable of this study was body mass index (BMI) calculated from self-reported height and weight dichotomized into normal weight (< 25 kg/m^2^) or overweight (including obese, ≥ 25 kg/m^2^), according to the criterion of Japanese National Health and Nutrition Survey [13].

### Exposure variable

Exposure variable was calculated from levels of PA and screen time (ST). For PA the Short Version of International Physical Activity Questionnaire (IPAQ-SV) was used. Total time spent in vigorous-intensity PA, moderate-intensity PA, and walking was calculated and dichotomized into "sufficient PA" (≥ 150 minutes/week) or "insufficient PA" (< 150 minutes/week) based on public health guidelines [14]. Test-retest reliability and criterion validity of the Japanese version of IPAQ-SV have been validated [15]. For ST, participants reported their time spent in the following screen-related sedentary behaviors: watching television, videos, and DVDs; Internet use (except work related); and video game use. Reasonable validity and reliability were reported [16]. The sum of the time spent in these behaviors was dichotomized into "low ST" or "high ST" using the median (21 hours/week). According to the levels of PA and ST, participants were classified into the following four categories: sufficient PA/low ST; sufficient PA/high ST; insufficient PA/low ST; and insufficient PA/high ST.

### Sociodemographic variables

Data on participants' sex, age (30-39; 40-49; 50-59 years), marital status (married; unmarried), educational level (junior high and high school degree; two-year college degree or equivalent; four-year college or higher degree), job status (full-time job; not full-time job), and household income (less than 5 million yen; 5-10 million yen; more than 10 million yen) were obtained from the research company.

### Data analysis

Data for 2832 adults who provided complete information for the study variables were analyzed. Logistic regression was conducted to estimate the odds ratios (ORs) of being overweight by PA/ST categories adjusted for sociodemographic variables. The sufficient PA/low ST category was the reference for this analysis. Regression analyses were performed for the whole sample, and separately for men and women, based on the gender differences have previously been found [8]. Analysis was conducted using SPSS 15.0; the level of significance was set at *p *< 0.05.

## Results

Table 1 shows the sociodemographic characteristics of the total sample and the four PA/ST categories. Overall, 50% of the respondents were men and 74.5% were married. Of the total respondents, 33.6% were aged 30-39 years, 33.3% were aged 40-49 years, and 33.1% were aged 50-59 years. In addition, 47.6% of the participants had graduated from college or graduate school and 62.0% had full-time job, as well as 46.5% had 5-10 million yen income. The proportion of overweight and obese participants was 21.8% for the total sample. Correlation coefficient between PA and ST was -0.17, suggesting that these two behaviors were essentially independent from each other.

**Table 1:** Sample characteristics by Physical Activity (PA) and Sitting Time (ST) Categories

		Combined Categories of PA and ST				
			
	Total	Sufficient PA/Low ST	Sufficient PA/High ST	Insufficient PA/Low ST	Insufficient PA/High ST	*p*-value
**No. (%)**	2832	905(32%)	656 (23%)	702 (25%)	569 (20%)	-
**Sex**						< 0.001
Male	50.0%	57.5%	44.7%	50.7%	43.4%	
Female	50.0%	42.5%	55.3%	49.3%	56.6%	
**Age group**						< 0.001
30-39 years	33.6%	35.6%	27.7%	38.0%	31.6%	
40-49 years	33.3%	29.8%	34.3%	36.8%	33.4%	
50-59 years	33.1%	34.6%	38.0%	25.2%	35.0%	
**Marital status**						< 0.001
Unmarried	25.5%	20.8%	31.3%	24.5%	27.6%	
Married	74.5%	79.2%	68.8%	75.5%	72.4%	
**Educational level**						< 0.001
Junior high/high school	26.4%	18.6%	33.5%	25.8%	31.5%	
Two-year college	26.0%	24.7%	25.0%	25.1%	30.4%	
Four-year college/graduate school	47.6%	56.7%	41.5%	49.1%	38.1%	
**Job status**						< 0.001
Full-time job	62.0%	68.3%	53.7%	69.8%	52.2%	
No full-time job	38.0%	31.7%	46.3%	30.2%	47.8%	
**Household income (yen p.a)**						< 0.001
< 5 million	38.0%	31.3%	41.6%	36.9%	45.9%	
5-10 million	46.5%	50.4%	42.8%	48.1%	42.7%	
> 10 million	15.5%	18.3%	15.6%	15.0%	11.4%	
**BMI**						0.182
Normal weight	78.2%	79.4%	79.1%	78.3%	74.9%	
Overweight	21.8%	20.6%	20.9%	21.7%	25.1%	
Mean BMI, kg/m^2 ^(sd)	22.59 (3.53)	22.46 (3.25)	22.51 (3.49)	22.52 (3.50)	22.99 (4.02)	

Abbreviations: PA: physical activity; ST: screen time, p.a.: per annum.

Table 2 shows ORs for being overweight or obese by combined categories of PA and ST for the total sample, for men, and for women, adjusting for sociodemographic factors. In the total sample, adults who were insufficient in PA and high in ST were 1.48 times more likely overweight compared with those who engaged in sufficient PA and were low in ST. The other categories (sufficient PA/high ST, insufficient PA/low ST) were not significantly different from the reference category. A similar pattern was observed in men. Compared with the reference category, men who engaged in insufficient PA and high ST were significantly more likely overweight. However, no significant association was found between the PA/ST categories and overweight in women.

**Table 2:** Adjusted Odds Ratios of Overweight/Obese by Physical Activity (PA) and Sitting Time (ST) Categories

	Being overweight (BMI, ≥ 25 kg/m^2^)					
	
	Total (n = 2832)		Men (n = 1416)		Women (n = 1416)	
	
	OR (95% CI)	*p*-value	OR (95% CI)	*p*-value	OR (95% CI)	*p*-value
Sufficient PA/low ST	1.00 (ref.)	-	1.00 (ref.)	-	1.00 (ref.)	-
Sufficient PA/high ST	1.13 (0.87-1.46)	0.37	1.30 (0.94-1.79)	0.11	0.87 (0.55-1.40)	0.57
Insufficient PA/low ST	1.12 (0.87-1.43)	0.39	1.18 (0.88-1.59)	0.28	0.97 (0.61-1.55)	0.91
Insufficient PA/high ST	1.48 (1.14-1.93)	0.003	1.50 (1.08-2.09)	0.02	1.43 (0.92-2.23)	0.11

OR adjusted for sex (whole sample), age, marital status, educational level, job status, and household income.

## Discussion

Japanese adults who engaged in insufficient PA and high ST were about 1.5 times more likely overweight than those with sufficient PA and low ST. Given that insufficient PA or high ST alone was not significantly associated with overweight, our findings suggest that it is the combination of lack of PA and prolonged ST that increases the risk of overweight and obesity in this sample of Japanese adults. This finding on the combined effect of PA and ST on overweight is consistent with a previous study that examined the joint association in the same manner [8]. However, our findings are different from that study in that we found the OR of being overweight was not significantly higher for sufficient PA/high ST and insufficient PA/low ST categories. This is also inconsistent with previous studies that demonstrated associations of sedentary time with obesity measures independent of physical activity [6,7]. The inconsistency between this and previous studies may stem from behaviors that were not measured in this study such as nonscreen-based sedentary behaviors (e.g., during work and transport) and light-intensity physical activity. The latter has been shown associated with reduced metabolic risk independent of moderate-to-vigorous physical activity [17]. It is possible that those in the sufficient PA/high ST category may be low in nonscreen-based sedentary behaviors (they may afford high ST in their leisure time due to less time commitment for work or transport), and those in the insufficient PA/low ST category may be high in light-intensity activity (they may have to cut PA and ST to perform duties such as household chores). Our findings suggest potentially different behavioral mechanisms linking physical activity and sedentary behavior with metabolic risk between Japan and Western countries, where previous studies have been conducted. Further research using objective behavioral measures is warranted to explore such differences.

In this study, significant associations between combined PA/ST categories and likelihood of being overweight or obese were found in men but not in women. This pattern of sex difference diverges from the findings of studies conducted in Australia [18], Europe [19], and the United States [20], which have shown stronger associations between sedentary behavior and metabolic health risks in women. One possible explanation is that the prevalence of overweight or obesity is very low among women in this sample. It was 12.5%, which is even lower than the national prevalence for women reported in the Japanese National Health and Nutrition Survey (20%) [21]. It is possible that women in this sample, particularly those who are not very active, may pay close attention to diet so as to control their weight. Future studies should examine diet so as better to understand obesity risks among Japanese women.

Several limitations need to be considered. First, the study used a cross-sectional design; thus it is not possible to make causal inferences. Second, the utilization of IPAQ-SV may cause the overestimation of PA time due to recall bias [22,23]. Third, ST was not measured separately by weekday and weekend, which may contribute to an inaccurate estimation of ST. Fourth, as discussed above, potentially confounding behaviors such as light-intensity activity and diet were not assessed in the study. Finally, the study sample was extracted from the list held by an Internet survey company. Previous studies have indicated that respondents to Internet-based surveys are generally younger, better educated, have higher income and may have greater access to the Internet than respondents to traditional surveys [24,25]. Thus the findings obtained from our sample may not be representative for the entire adult population of Japan.

Regardless of the limitations, our findings suggest the importance of addressing both aspects of physical inactivity (insufficient PA and high ST) to reduce overweight and obesity at the population level. Future health promotion strategies addressing obesity in Japan should focus not only on increasing PA but also on reducing sedentary time, especially in men.

## Competing interests

The authors declare that they have no competing interests.

## Authors' contributions

YL contributed to analysis and interpretation of data and drafted and revised the paper. KH participated in the study design, contributed to analysis and interpretation of data, and revised the paper. AS, KI, KO, SI conceived the study, participated in its design and coordination, and helped in drafting the manuscript. TSU, YN, TSH performed the sequence alignment and helped in drafting the manuscript. All the authors have read and approved the final manuscript.
